# Detection of suicide risk using event-related potentials: a comprehensive systematic review and meta-analysis

**DOI:** 10.1093/psyrad/kkaf018

**Published:** 2025-06-11

**Authors:** Qianlan Yin, Huijing Xu, Zhuyu Chen, Qian Jiang, Taosheng Liu

**Affiliations:** Department of Psychiatry, Faculty of Psychology and Mental Health, Naval Medical University, Shanghai, 200433, China; Department of Psychiatry, Faculty of Psychology and Mental Health, Naval Medical University, Shanghai, 200433, China; Department of Psychiatry, Faculty of Psychology and Mental Health, Naval Medical University, Shanghai, 200433, China; Department of Psychiatry, Faculty of Psychology and Mental Health, Naval Medical University, Shanghai, 200433, China; Department of Psychiatry, Faculty of Psychology and Mental Health, Naval Medical University, Shanghai, 200433, China

**Keywords:** electroencephalography (EEG), event-related potentials (ERPs), suicide risk, P3, cognitive processing, neural indicators

## Abstract

**Background:**

Suicide has profound effects on individuals, families, and societies globally, underscoring the urgent need for effective early detection and prevention strategies. This systematic review aims to investigate the use of event-related potentials (ERPs) as a tool for identifying and monitoring suicide risk.

**Methods:**

A comprehensive literature search was conducted, resulting in the inclusion of 23 articles that met the eligibility criteria. The review synthesized findings related to various ERP components associated with suicide risk.

**Results:**

The analysis revealed that individuals with a history of suicide risk exhibited significantly reduced P3 amplitudes in response to novel stimuli during the go/no-go paradigm compared to healthy controls [standardized mean difference (SMD) = −0.53, 95% confidence interval (CI) = [−0.96; −0.10]]. Additionally, altered P3 responses to positive feedback on rewards indicated impairments in those at risk (SMD = −1.12, 95% CI = [−1.74; −0.49]). Variability in other ERP components was also highlighted, with several moderators, such as sample characteristics and methodological design, influencing ERP components.

**Conclusion:**

The findings suggest that specific ERP components, particularly the P3, may serve as valuable indicators for assessing suicide risk. The review emphasizes the need for future research to utilize larger, more homogeneous samples and advanced analytical techniques to enhance detection accuracy. The application of ERPs is posited as a promising avenue for improving understanding of the neurocognitive mechanisms associated with suicide risk and enhancing prevention efforts.

## Introduction

Suicide has a universal impact, transcending geographical and age boundaries to affect individuals and families globally. With an approximate ratio of 20 suicidal attempts to every reported death, suicide emerges as a significant contributor to mortality rates, particularly among young people (WHO, 2022). The prevalence of suicidal underscores the urgent need for effective early detection and prevention strategies.

Suicide risk is characterized by the potential for individuals to engage in self-harm with the intent of ending their life (Jans *et al*., 2012). It encompasses both suicidal ideation (SI)—thoughts or plans about self-harm, including active ideation (explicit thoughts of engaging in self-harming behaviors with the intent to end one's life) and passive ideation (desires to die or thoughts about death without a specific plan or intent to act)—and suicidal behavior, which includes actual attempts or completed suicides. Suicide risk can be affected by a range of factors, such as psychological distress and environmental influences, as well as cognitive factors (Turecki *et al*., 2019). More specifically, SI can be intensified by distorted thinking, negative self-perception, hopelessness and lack of belonging, leading to an increased risk of attempts (Shira and Alan, 2014). It is crucial to understand that SI can manifest in different ways, from fleeting thoughts to detailed plans, while a suicidal attempt (SA) involves deliberate actions with the intent to die (Sun *et al*., 2023; Saadabadi, 2024). However, both SI and attempts are part of the spectrum of suicidal behaviors (Sun *et al*., 2023). They share common underlying factors of suicide risk (Nock *et al*., 2010). In particular, impaired decision-making and impulsivity, as cognitive factors, can further facilitate the progression from SI or attempts to actions (Yari *et al*., 2015). Consequently, identifying early the contributing cognitive factors on suicide could be essential in mitigating the potential risk.

Research conducted over the past few decades has delved into the intricate and multifaceted factors of suicide risk. However, the current approaches to suicide risk research have limitations. These methods, which rely primarily on self-reported, demographic and historical factors, often have poor diagnostic accuracy despite being statistically significant predictors of future suicidal behaviors (Franklin *et al*., 2017). Recent advances in neurophysiology have provided promising avenues for objective risk detection. In particular, electroencephalography (EEG) and its derived measure, event‐related potentials (ERPs), offer a non-invasive method to probe the neural substrates underlying cognitive and affective processes that may predispose individuals to suicidal behavior (Gibb and Tsypes, 2019; Song *et al*., 2019). Over the years, the number of publications addressing the intersection of suicide and EEG research has grown remarkably. This expanding body of literature underscores the increasing recognition of ERP methods as a tool for elucidating the neural underpinnings of suicide risk.

ERPs represent time-locked electrical responses of the brain to specific sensory, cognitive, or motor events. With millisecond temporal resolution, ERPs provide unique insights into the rapid neural processes that underlie perception, attention, memory, and decision-making. The utility of ERPs as neurophysiological biomarkers, has been demonstrated in a wide range of applications—from the detection of deception to risk assessments in populations vulnerable to substance abuse (Marvi *et al*., 2023; Rahimi Saryazdi *et al*., 2024). More recently, studies have focused on identifying ERP components that are specifically associated with suicide risk. For example, Gibb and Tsypes proposed that certain ERP measures can improve the prediction of suicide risk by revealing deficits in cognitive-emotional processing that are not captured by self-report measures (Gibb and Tsypes, 2019). The rationale for using ERP-based approaches in suicide risk detection is multifaceted. First, traditional methods of suicide risk assessment rely heavily on subjective clinical interviews and self-report questionnaires. Although these tools are important, they are often limited by issues such as social desirability bias and patients’ inability to self-report accurately, particularly in states of acute emotional distress. ERPs, on the other hand, provide an objective, quantifiable measure of neural activity. They are less susceptible to such biases and can capture subtle alterations in brain function that precede overt behavioral manifestations of suicidal behavior (Albanese *et al*., 2019). Second, several ERP components have been implicated in the processes that are critical to understanding suicidal behavior. Among these, the P300, N400, error-related negativity (ERN), late positive potential (LPP), and feedback-related negativity (FRN) provide valuable insights into the neurocognitive mechanisms associated with suicide risk. Specifically, the P300 component is reflective of attention, memory, and decision-making, while the N400 is associated with semantic processing and language comprehension (Yang *et al*., 2012). The ERN component plays a role in error detection and cognitive control, and the LPP is linked to emotional processing and regulation (Wauthia and Rossignol, 2016). Additionally, the FRN is related to reward processing and decision-making (Liu *et al*., 2015). Together, these ERP measures can enhance clinical identification and assessment of suicide risk by objectively evaluating cognitive processes such as attention, emotional processing and impulse control (Campanella, 2021).

Accordingly, this review endeavors to synthesize the components of the cognitive processes and neurocognition of suicide to provide a comprehensive understanding of the utilization of ERP in detecting suicide risk. The primary aim of our review was to synthesize the existing evidence and identify a set of robust neural indicators that can facilitate the objective evaluation of suicide risk.

## Methods

### Search strategy

A comprehensive literature review was undertaken across the PubMed, Embase, and PsycINFO databases to identify relevant studies published up to 1 December 2024. A search strategy based on the following key search terms was used to identify the relevant literature: (“suicide” OR “suicidal behavior” OR “suicide risk” OR “suicidal ideation” OR “suicidal attempts” OR “self-injurious behavior” OR “self-harm” OR “self-mutilation” OR “self-inflicted injury” OR “suicidal thoughts” OR “completed suicide”) AND (“EEG” OR “electroencephalography”). Truncations and related terms were used as appropriate based on individual database procedures (see Appendix 1 for the full search strategy). The search was last updated on 4 December 2024.

Studies were included/excluded in this review if they met the following criteria:

### Inclusion criteria

1. Population: studies involving individuals below 60 years of age with SI, SA, or a high risk of suicide. This may include individuals diagnosed with depression, bipolar disorder, or other psychiatric conditions associated with suicidality.
2. Intervention/comparison: studies that utilize ERPs as a neuroimaging technique. This may involve comparing ERP data from individuals at suicide risk to healthy controls or individuals with other psychiatric conditions.
3. Outcome: studies that investigate specific ERP components associated with cognitive processes relevant to suicide risk, such as attention bias, emotional processing, and error monitoring.
4. Study design: studies with robust methodological designs, such as randomized controlled trials, pre-post studies, or well-controlled observational studies.
5. Publication language: articles published in English to ensure comprehensibility and avoid language bias.

### Exclusion criteria

1. Population: not specifying participant age.
2. Intervention/comparison: ERP was not used as a neuroimaging technique.
3. Outcome: did not measure or report on any ERP components.
4. Study design: these were not published as peer-reviewed journal articles.

### Data extraction

Data were extracted from the included studies by two independent reviewers (X.H. and C.Z.), with any discrepancies resolved through discussion or consultation with a third reviewer (Y.Q.). The data extracted from the articles that underwent review for the purpose of the meta-analysis are accessible at https://osf.io/bdux8.

The following data extraction sequence was used: author, publication year, country, samples, study objectives, study design, sample size, participant distribution across study groups, age, gender distribution, diagnostic methodology, relevant psychological measures, task, ERP components examined, electrode locations, key findings, and quantitative ERP data for group comparisons. To obtain as much quantitative ERP data as possible, the corresponding authors were contacted via email when the data were not available or incomplete in the reviewed articles, and plot digitizer software was used to extract missing data from figures/plots. The characteristics of the included studies were manually extracted and tabulated to ensure accuracy.

### Quality assessment

Two authors (X.H. and C.Z.) independently evaluated the risk of bias in each study based on Version 2 of the Cochrane risk-of-bias tool for randomized trials (RoB2) (Sterne *et al*., 2019). J.Q. and L.T. did not participate in the RoB2 assessment, given that they authored papers included in the current systematic review. Y.Q. reviewed the data extraction and further discussion with X.H. and C.Z. Any discrepancies in the RoB were resolved by discussion. RoB2 is structured into a fixed set of domains of bias, focusing on different aspects of trial design, conduct, and reporting. Within each domain, a series of questions (“signaling questions”) aim to elicit information about features of the trial that are relevant to risk of bias. A proposed judgement about the risk of bias arising from each domain is generated by an algorithm, based on answers to the signaling questions. Judgement can be “Low” or “High” risk of bias or can express “Some concerns.”

The diverse array of studies included in the analysis posed difficulties in consolidating their respective limitations. In response, the review considered variables such as the variations in research methodologies, participant demographics, and the particular ERP elements under investigation. Qualitative analysis was conducted to identify common methodological issues, gaps in the current literature, and recommendations for future research to strengthen the application of ERP in detecting suicide risk. A general overview of the main characteristics and results is presented in Table 1. Further data synthesis and meta-analysis were applied to quantitative ERP data where feasible. Random-effects models were utilized, and study-level effect sizes were calculated as standardized mean differences with associated 95% confidence intervals. Notably, to accommodate the multigroup designs found in certain studies, we incorporated data from multiple participant groups within the same study into our analysis.

**Table 1:** The characteristics and results of the studies included in the systematic review.

Article	Country	*n*	Group	Sample	Age (years, mean ± SD)	Gender (female-male)	Diagnosis criterion
Chen *et al*., 2005	China	66	SG: Suicide group; NSG: No suicide group	NSG = 50/SG = 16	NSG = 25.6 ± 7.0/SG = 28.0 ± 5.3	NSG = 30–20/SG = 9–7	History of suicidal attempts
Jandl *et al*., 2010	Germany	50	HAS: “hard” attempted suicide group; SAS: “soft” attempted suicide group; NAS: non-attempted suicide group	HAS = 16/SAS = 16/NAS = 18	HAS = 47.3 ± 11.9/SAS = 49.8 ± 8.0/NAS = 47.9 ± 11.6	HAS = 9–7/SA = 11–5/NAS = 12–6	History of suicidal attempts
Marsic *et al*., 2015	USA	41	NSIB: Non-suicidal self-injurious behavior; SIB: Suicidal self-injurious behavior	NSIB = 11/SIB = 30	20.69 ± 2.98	All men	Deliberate Self-Harm Inventory
Kudinova *et al*., 2016	USA	33	SIG: Suicidal Ideation group; NSIG: No Suicidal Ideation group	SIG = 10/NSIG = 23	SIG = 18.70 ± 1.06/NSIG = 20.22 ± 2.31	SIG = 9–1/NSIG = 14–9	Mini-International Neuropsychiatric Interview
Weinberg *et al*., 2017	USA	235	SA: History of suicidal attempts; NSA: No suicidal attempts	SA = 83/NSA = 152	SA = 40.22 ± 11.81/NSA = 41.70 ± 13.64	HC = 42–41/NSA = 87–65	Structured Clinical Interview for DSM-5
Albanese *et al*., 2019	USA	68	SA: History of suicidal attempts; NSA: No past suicidal attempts	SA = 22/NSA = 46	SA = 42.91 ± 16.09/NSA = 33.63 ± 14.45	HC = 13–9/NSA = 21–25	Structured Clinical Interview for DSM-5
Tsypes *et al*., 2019	USA	69	RSI: Recent suicide ideation group; NRSI: No recent suicide ideation group	RSI = 23/NRSI = 46	RSI = 9.23 ± 1.33/NRSI = 9.64 ± 1.25	HC = 9–14/NRSI = 25–21	Schedule for Affective Disorders and Schizophrenia
Song *et al*., 2019	China	56	HC: Healthy controls; MDD-HSR/LSR: Major depression disorder with high/low risk of suicide	HC = 24/MDD-HSR = 23/MDD-LSR = 9	HC = 20.75 ± 2.95;MDD-HSR = 24.57 ± 5.69;MDD-LSR = 23.33 ± 5.32	HC = 13–11/MDD- HSR = 15–8/MDD- LSR = 4–5	Structured Clinical Interview for DSM-5
Song *et al*., 2020	China	72	HC: Healthy controls; MDD-SI/SA: Major depression disorder with suicidal ideation/attempts	HC = 28/MDD-SI = 32/MDD-SA = 12	HC = 20.54 ± 3.13/MDD-SI = 23.72 ± 4.52/MDD-SA = 24.58 ± 7.19	HC = 16–12/MDD-SI = 19–13/MDD-SA = 10–2	Structured Clinical Interview for DSM-5
Kim *et al*., 2021	Korea	130	HC: Healthy controls; MDD-SI/SA: Major depression disorder with suicidal ideation attempts	MDD-SA = 45/MDD-SI = 49/HC = 36	MDD-SA = 32.07 ± 9.49/MDD-SI = 32.06 ± 9.34/HC = 31.44 ± 5.20	HC = 25–20/MDD-SI = 26–23/MDD-SA = 17–19	Structured Clinical Interview for DSM-5
Tavakoli *et al*., 2021	Canada	28	ASB: Adolescents with acute suicidal behavior group; HC: Healthy control group	ASB = 14/HC = 14	ASB = 15.7 ± 1.1/HC = 15.7 ± 1.1	HC = 9–5/ASB = 9–5	Structured Clinical Interview for DSM-5
Tsypes *et al*., 2021	USA	60	SA: Suicidal attempts; NSA: Non-suicidal attempts	SA = 30/NSA = 30	SA = 23.23 ± 8.13/NSA = 26.60 ± 12.36	HC = 23–7/NSA = 19–11	History of suicidal attempts
Yoon *et al*., 2022	Korea	150	SA: Suicidal attempts; SI: Suicide ideation	SA = 74/SI = 76	SA = 39.38 ± 14.31/SI = 41.53 ± 14.78	SA = 43–31/SI = 46–30	Experiments of being admitted to hospital
Camsari et al. 2023	USA	57	SIBS: Adolescents with suicidal ideations and behaviors; HC: Healthy control	SIBS = 27/HC = 30	SIBS = 15.75 ± 13.4/HC = 15.475 ± 18.3	SIBS = 21–6/HC = 16–14	Mini-International Neuropsychiatric Interview
Kim *et al*., 2023	Korea	162	HC: Healthy control; NSI: MDD without suicide ideation; SI: MDD with suicide ideation	HC = 27/NSI = 31/SI = 104	HC = 28.89 ± 5.67/NSI = 45.1 ± 13.4/SI = 35.8 ± 16.5	HC = 13–14/NSI = 21–10/SI = 63–41	Beck Scale for Suicidal Ideation
Liu *et al*. 2023	China	74	SA: Suicide attempt; NSA: Patients with mental disorders without past suicidal attempts; HC: Healthy control	SA = 26/NSA = 26/HC = 22	SA = 36.88 ± 15.37/NSA = 39.42 ± 11.21/HC = 43.36 ± 14.07	SA = 18–8/NSA = 14–12/HC = 16–6	A Chinese version of the Mini-International Neuropsychiatric Interview
Porteous *et al*., 2023	Canada	24	HC: Healthy control; SA: Suicide attempt	HC = 12/SA = 12	HC = 14.42 ± 1.2/SA = 15.08 ± 1.5	HC = 8–4/SA = 9–3	Criteria from the DSM-5
Song *et al*., 2023	China	72	MDD-SI: MDD patients with suicide ideation; MDD-SA: MDD patients with suicide attempt; HC: Healthy control	HC = 28/MDD-SI = 32/MDD-SA = 12	HC = 20.54 ± 3.13/MDD-SI = 23.72 ± 4.52/MDD-SA = 24.58 ± 7.19	HC = 16–12/MDD-SI = 19–13/MDD-SA = 10–2	History of suicidal attempts
Sun *et al*., 2023	China	44	SIMDs: Suicidal ideation with mental disorders; SINMDs: Suicidal ideation without mental disorder; HC: Healthy control	SIMDs = 14/SINMDs = 16/HC = 14	SIMDs = 20.36 ± 2.44/SINMDs = 18.94 ± 1.57/HC = 21.00 ± 2.11	SIMDs = 6–8/SINMDs = 5–11H/C = 7–7	A Chinese version of the Mini-International Neuropsychiatric Interview
Klumpp *et al*., 2023	USA	109	SI: Suicide ideation group; NSI: Non-suicide ideation group	SI = 59/NSI = 50	SI = 26.10 ± 8.19/NSI = 29.98 ± 10.03	SI = 37–22/NSI = 37–13	Self-report Inventory of Depression and Anxiety Symptoms
Bao *et al*. 2024	China	119	SA: Suicide attempt group; NSA: MDD with no suicidal attempt group; HC: Healthy group	SA = 39/NSA = 40/HC = 40	SA = 21.23 ± 3.80/NSA = 21.02 ± 2.87/HC = 22.23 ± 2.29	SA = 21–18/NSA = 18–22/HC = 27–13	A Chinese version of the Mini-International Neuropsychiatric Interview
Li *et al*. 2024	China	118	MDD-SA: MDD with a history of suicidal attempts; MDD-NSA: MDD without suicide attempt; HC: Healthy control	MDD-SA = 32/MDD-NSA = 48/HC = 38	MDD-SA = 20.16 ± 4.03/MDD-NSA = 23.19 ± 4.55/HC = 21.37 ± 5.79	MDD-SA = 25–7/MDD-NSA = 31–17/HC = 20–18	History of suicidal attempts
Zhou *et al*., 2024	China	258	HC: Healthy group; MDD-low: MDD in low suicidal risk; MDD-Moderate: MDD in moderate suicidal risk; MDD-High: MDD in high suicidal risk	HC = 70/MDD-low = 56/MDD-Moderate = 66/MDD-High = 66	HC = 38.2 ± 13.3/MDD-low = 43.2 ± 11.5/MDD-Moderate = 40.6 ± 11.8/MDD-High = 38.5 ± 13.2	HC = 49–21/MDD-low = 44–12/MDD-Moderate = 45–21/MDD-High = 53–13	Nurse's Global Assessment of Suicide Risk

## Results

### Literature search and characteristics of studies included in analyses

**Figure 1:**
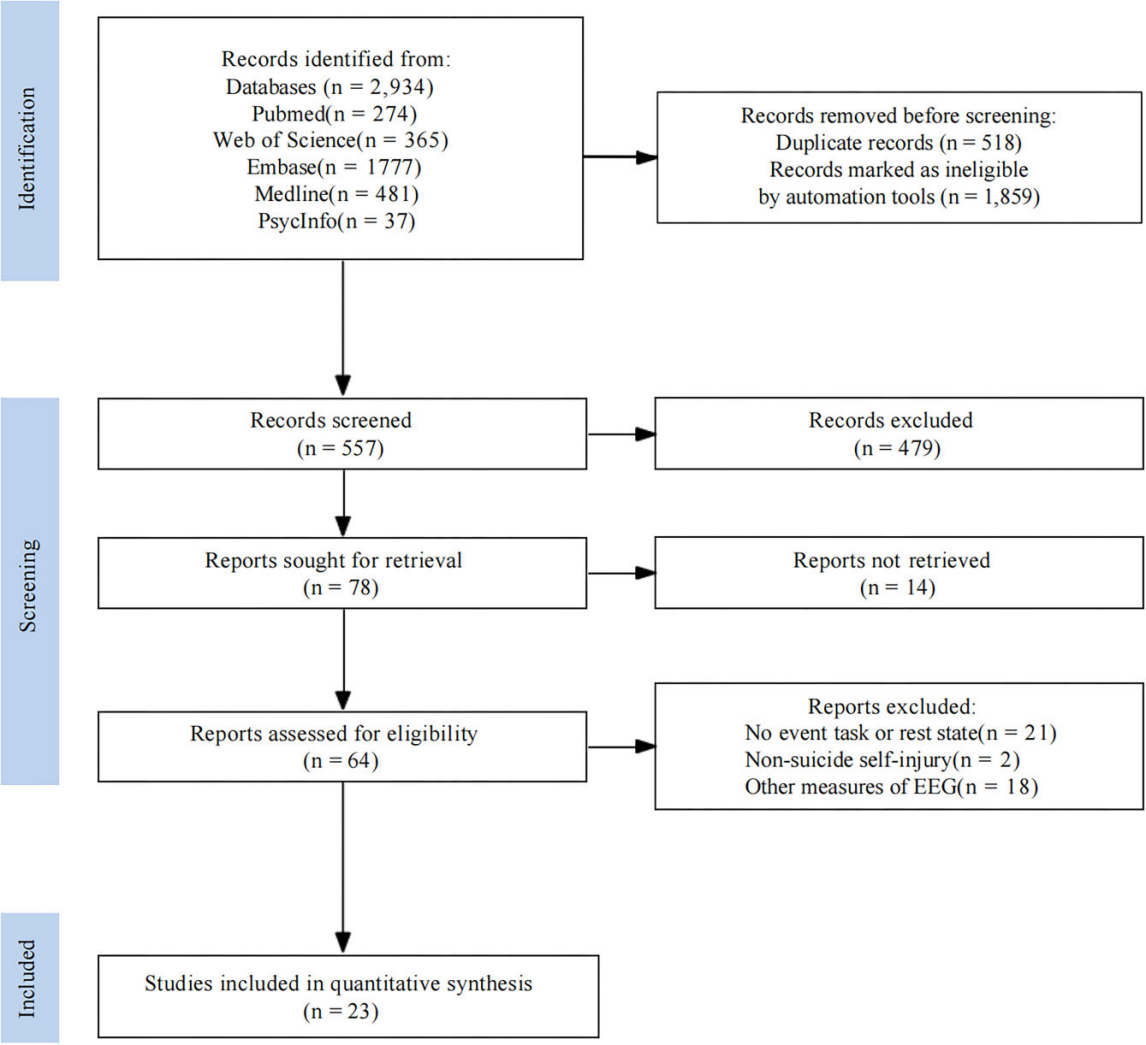
Flow diagram following the Preferred Reporting Items for Systematic Reviews and Meta-analysis guidelines.

The PRISMA flow diagram illustrates the systematic review process for selecting studies (Fig. 1). A total of 2934 records were identified from various databases, including PubMed (*n* = 274), Web of Science (*n* = 365), Embase (*n* = 1777), Medline (*n* = 481), and PsycInfo (*n* = 37). After removing 518 duplicates and 1859 records marked as ineligible by automation tools, 557 records were screened according to predefined criteria. Of these, 479 were excluded for not meeting the inclusion criteria, leaving 78 reports to be retrieved. Fourteen reports could not be retrieved, and 64 were assessed for eligibility. Among these, 41 were excluded due to either lacking event tasks or rest-state measures (*n* = 21), or using other EEG measures (*n* = 18). It is worth noting that the present review did not encompass studies on non-suicidal self-injury (*n* = 2), as it was outside the defined scope of this investigation. Consequently, one relevant article was excluded from the analysis. Finally, 23 studies met the criteria and were included in the quantitative synthesis, reflecting a rigorous selection process to ensure relevance and quality. Most of these studies were conducted in the USA (*n* = 9), followed by China (*n* = 8), Korean (*n* = 2), and other countries (*n* = 4). The number of studies in this area has been increasing in recent years. The majority of the included studies employed a cross-sectional design and investigated differences in ERP components between individuals at suicide risk and healthy control participants. Some studies categorized participants based on high versus low levels of suicide risk, employing a distinction between “hard” and “soft” suicide risk, while some grouped according to the distinction between SI and SA (Jandl *et al*., 2010). To summarize, the reviewed studies cover a range of research questions, study designs, and ERP markers relevant to suicide risk.

### Participants

The reviewed studies encompassed 2039 individuals, with sample sizes varying from 24 to 258 participants. The characteristics and outcomes of the included studies are outlined in Table 1 for reference in the systematic review. Among these cohorts, 510 individuals were identified as having suicide risk. Of the 13 studies, some included participants with SA, while others included SI or both. Only one study directly focused on suicide risk. The majority of the studies had small sample sizes, while only a few had larger numbers of participants. The age range of participants was from 10 to 60 years, with two studies involving teenagers and one study concentrating on individuals aged 50 years and older. Most studies included both male and female participants. The studies commonly utilized clinical interviews and assessments to diagnose and classify participants' SI and SA. The reviewed studies also utilized various standardized emotional and behavioral assessment measures, such as the Suicide Probability Scale, Beck Depression Inventory, Beck Suicide Ideation Scale, and Behavioral Impulsivity Scales, to further characterize the study participants. Most of the studies involved participants with co-occurring depression, which is a significant risk factor for suicide.

### Qualitative and quantitative analysis of the selected studies

The reviewed studies utilized various ERP paradigms to investigate different cognitive processes related to suicide risk. As presented in Table 2, these included oddball tasks (attentional processing), go/no-go tasks (inhibitory control), emotional Stroop tasks (emotion processing), and other task-related paradigms focused on the processing of reward such as the Doors task and Monetary Incentive Delay (MID) task. The auditory oddball paradigm is the most frequently utilized task across the reviewed studies. A total of six studies employed this paradigm (Chen *et al*., 2005; Jandl *et al*., 2010; Kudinova *et al*., 2016; Kim *et al*., 2021; Tavakoli *et al*., 2021; Yoon *et al*., 2022). The go/no-go task is another commonly used paradigm in the ERP studies of suicide risk with three studies employing it (Albanese *et al*., 2019; Kim *et al*., 2023; Porteous *et al*., 2023). Furthermore, the image-viewing tasks, predominantly utilizing a passive form to examine the neural connections of emotional processing in individuals with different levels of suicidal thoughts, are utilized to highlight different aspects of cognitive and emotional processing (Kudinova *et al*., 2016; Weinberg *et al*., 2017).

**Table 2:** A comprehensive overview of the ERP components and results from each study.

Article	Tasks	Components	Results
Chen *et al*., 2005	An auditory oddball paradigm	N1, P2, P3	SA showed a sharper slope of the LDAEP and increased frontal P3 amplitude.
Jandl *et al*., 2010	An auditory oddball paradigm	P3	P3 decrease over time was significantly more pronounced in both HAS and SAS when compared to NAS.
Marsic *et al*., 2015	An auditory oddball paradigm	N1, P2	The LDAEP index was significantly related to the most lethal form of self-injury.
Kudinova *et al*., 2016	An image-viewing task	LPP	SI had significantly higher LPP when asked to reduce negative emotion in response to dysphoric images.
Weinberg *et al*., 2017	An image-viewing task	LPP	SA exhibited a blunted threat-elicited LPP.
Albanese *et al*., 2019	A go/no-go Complex Paradigm	N2, P3	In terms of latent N2, the SA group in no-go trials was more positive than that of the no-SA group.
Tsypes *et al*., 2019	Guessing Doors task	ΔRewP	SI children exhibited significantly smaller ΔRewP than children with no SI.
Song *et al*., 2019	Affective Incentive Delay (AID) task	P2, P3	Feedback-P3 may be an electrobiological component underlying the processing of psychological pain in suicidality.
Song *et al*., 2020	Monetary Incentive Delay (MID) task	P3	The P3 elicited by negative feedback under punitive conditions was significantly larger than those of reward and neutral conditions in the MDD-SA group, whereas no significant differences were found between the MDD-SI and HC groups.
Kim *et al*., 2021	Auditory evoked potential task	N1, P2	MDD patients with SA and SI had significantly weaker LDAEP than healthy controls at the Cz electrode, but the significance disappeared when clinical variables such as BDI, BAI, BIS, and DERS were controlled.
Tavakoli *et al*., 2021	An auditory oddball paradigm	N2, P3	A slight, but non-significant, increase in the amplitude of the novel-N2 and -P3 in the suicidal group.
Tsypes *et al*., 2021	Monetary Incentive Delay (MID) task	P3, FRN	SA exhibited blunted the cue-related P3.
Yoon *et al*., 2022	An auditory oddball paradigm	N2, P3	SA had significantly decreased no-go P3 amplitudes at all electrodes.
Camsari *et al*., 2023	An visual oddball paradigm task	N1, P2, LPP	Adolescents with SI had larger N1 during death-congruent blocks than life-congruent blocks, whereas this difference was reversed for HCs.
Kim *et al*., 2023	The go/no-go tasks	N2, P3	The MDD with SI showed significantly decreased Nogo P3 amplitudes compared to MDD without SI.
Liu *et al*. 2023	Ultimatum game	P2, FRN	SA had decreased P2 amplitude and prolonged P2 latency when receiving unfair offers. Moreover, SA patients exhibited greater negative-going FRN to unfair offers compared to fair ones, whereas such a phenomenon was absent in NSA and HC groups. These results revealed that SA patients had a stronger fairness principle and a disregard toward the cost of punishment in social decision-making.
Porteous *et al*., 2023	A emotional version of go/no-go task	N2, P3	SA showed significantly reduced P3d (difference waveform reflecting no-go minus go trials) in response to happy and neutral, but not sad stimuli.
Song *et al*., 2023	Monetary Incentive Delay (MID) task	P3	The continued negative variation along with pain avoidance elicited by punitive cues may be a biomarker in suicide ideation.
Sun *et al*., 2023	A modified emotional Stroop task	N2, P3	SIMD group exhibited longer early posterior negativity (EPN) latency; P3 latency for positive words was positively correlated with current SI in the SINMD group.
Klumpp *et al*., 2023	Doors task	ΔRewP, FRN	ΔRewP was found to be lower in the SI; SI group showed less FRN.
Bao *et al*. 2024	The Iowa Game Task	FRN, P3	SA group and NSA exhibited blunted ΔFRN.
Li *et al*. 2024	The modified Affective Delay (AID) task	P3, LPP, FRN, P2	Reward feedback was the related neural representation in suicide attempt.
Zhou *et al*., 2024	Mismatch negativity (MMN) paradigm	N1, N2, P2, P3, FRN, P50, P3	MDD patients at moderate or high risk of suicide showed significant difference with HCs in the amplitude of P2–P3.

Note: ERP components elicited in each paradigm are categorized based on their polarity (positive or negative) and latency (in 100-ms units) relative to stimulus presentation. RewP denotes Reward Positivity. In the Doors Task context, ΔRewP represents the difference in RewP between receiving a reward and not receiving a reward/loss. LPP, Late Positive Potential; BDI, Beck Depression Inventory; BAI, Beck Anxiety Inventory; BIS, Barratt Impulsiveness Scale; DERS, Difficulties in Emotion Regulation Scale.

Table 2 also provides a detailed summary of ERP components and outcomes from all studies. The early components, N1 and P2, consistently serve as indicators of early attentional and perceptual processing, evident in auditory oddball paradigms. The middle components such as the N2 component are assessed in go/no-go paradigms and connected to cognitive control and response inhibition; the P3 component emerges as a critical marker for cognitive processes associated with attention, decision-making, and memory across various tasks, including the auditory oddball and monetary incentive delay tasks. Similar temporal characteristics to N2 and P3, FRN and Reward Positivity (RewP) measured by the MID task also provide an indication for suicide risk. Meanwhile, LPP is pivotal for understanding emotional processing, particularly in response to emotionally charged stimuli, as seen in image-viewing tasks. Collectively, these findings underscore the nuanced role of specific ERP components in elucidating cognitive and emotional dynamics related to suicide risk across diverse experimental contexts.

### The early-latency ERP components

**Figure 2:**
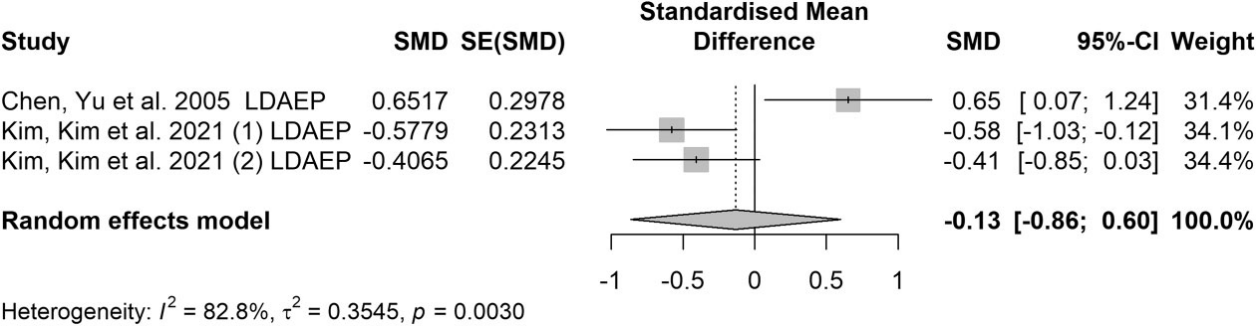
Forest plot of the LDEAP component elicited in the oddball tasks. Note: This forest plot illustrates the effect sizes (standardized mean difference, SMD) and 95% confidence intervals (CIs) for each study included in the meta-analysis. Each square represents the effect size for an individual study, with the size of the square corresponding to the weight of the study in the overall analysis. Horizontal lines extending from each square indicate the 95% CI for the effect size. The diamond at the bottom represents the overall effect size and its CI, summarizing the collective findings of the studies. The *x*-axis denotes the SMD, with a vertical line at zero indicating no effect of suicide risk. The studies are presented on the *y*-axis, illustrating the range of effect sizes among them and the overarching pattern suggesting a rise or fall in ERP components among individuals with suicidal inclination. Kim *et al*.’s study involved two group comparisons, labeled as (1) and (2).

The N1 or P1 component is sensitive to basic stimulus characteristics such as intensity and is believed to reflect early visual processing. However, limited research is available on how N1 may be altered in individuals at risk of suicide. Some studies suggest that individuals with SI or a history of SA may exhibit reduced N1 amplitudes in response to emotional stimuli, potentially indicating blunted early attentional engagement (Camsari *et al*., 2023; Zhou *et al*., 2024). Despite some inconsistencies in the findings across studies, the P1 component, akin to the N1, is a positive potential peaking around 100–200 ms post-stimulus, and is implicated in selective attention and discrimination of relevant stimuli (Kangas *et al*., 2022; Fischer-Jbali *et al*., 2024). However, research on changes in N1 and P1 related to suicide risk is limited, with most researchers advocating for the evaluation of changes in the amplitude of the evoked N1/P2 component in response to different intensities of the auditory stimulus, which is named as loudness dependence of the auditory evoked potential (LDAEP) (Chen *et al*., 2005; Marsic *et al*., 2015; Kim *et al*., 2021). It quantifies the relationship between the intensity of an auditory stimulus and the amplitude of the brain's electrical response. This measure is considered a reliable, non-invasive indicator of central serotonergic function. Lower serotonin levels are associated with a more pronounced increase in LDAEP as sound intensity increases, while higher serotonin levels are associated with a less pronounced increase in this amplitude–intensity relationship (Roser *et al*., 2020). The findings from the two previous studies that investigated the loudness dependence of the auditory evoked potential in individuals at risk of suicide have been inconsistent. Some studies have found that individuals with SI or SA are associated with a more pronounced decrease in LDAEP as sound intensity increases, compared to health controls. However, the significance disappeared when factors such as depression severity were controlled. There was no significant difference in LDAEP between MDD patients with SA and SI (*P* = 0.59). The specific findings across the studies examining LDAEP in depressed people with SA have been controversial, with inconsistent results across studies (Fig. 2). The high heterogeneity (*I*² = 82.8%) among the studies indicates substantial variation in the effects observed across different research. Egger's test revealed no publication bias (*b* = 15.89, *P* = 0.164; see Supplementary Fig. 2), as smaller studies with both significant and non-significant results were all included. The differences in samples may be a major reason for the discrepancies in the research on LDAEP.

### The middle-latency ERP components

**Figure 3:**
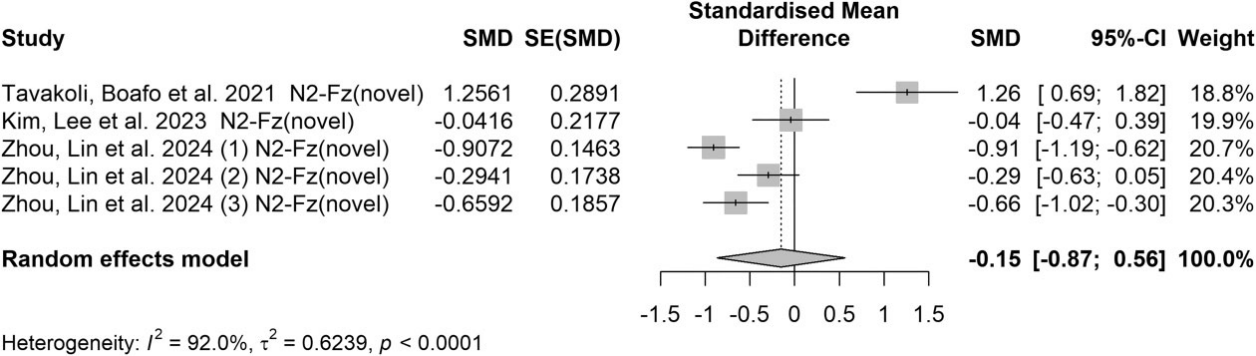
Forest plot of the N2 component elicited by novel cues at Fz in go/no-go studies. Note: The elements in this figure are identical to those in Fig. 1 with a consistent representation of comparisons across multiple groups in Zhou *et al*.’s investigation.

**Figure 4:**
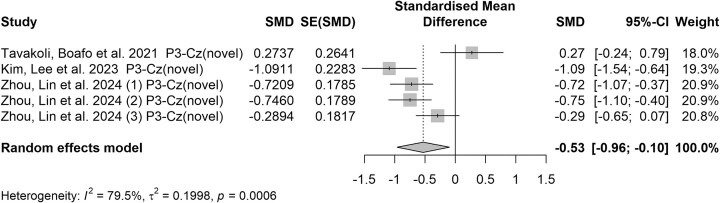
Forest plot of the P3 component elicited by novel cues in go/no-go studies. Note: The elements in this figure are identical to those in Fig. 1.

N2 and P3 are pivotal in studies utilizing the go/no-go paradigm. The N2 component has been associated with conflict detection and the recruitment of cognitive control to inhibit a response, while the P3 is linked to successful inhibition of the motor response. In our review, three studies used the go/no-go paradigm, showing that individuals with a history of SA exhibited altered N2 and P3 amplitudes compared to those with SI alone or healthy controls. Additionally, the study by Zhou *et al*. examined three levels of SA and compared each to healthy controls. The quantitative analysis incorporated three subgroups from Zhuo's study into the synthesis and indicates the N2 component exhibits more variability across studies, with some suggesting increased and others decreased amplitudes (see Fig. 3). On the other hand, negative effect sizes for the P3 components, signifying reduced amplitudes in individuals with suicidal tendencies compared to controls (SMD = −0.53, 95% CI = [−0.96; −0.10], see Fig. 4). This suggests a clear impairment in neurocognitive processing related to these individuals, reinforcing the P3 component as a reliable marker of suicidal risk. Importantly, high *I*² values (>50%) were observed in our pooled analysis for the N2 and P3 components, indicating considerable variability among the studies, probably due to diverse methodologies in task designs and differing participant demographics. Additionally, Egger's test revealed publication bias existed in N2 (*b* = 14.07, *P* = 0.014; see Supplementary Fig. 3) but not for P3 (*b* = 4.99, *P* = 0.532; see Supplementary Fig. 3).

**Figure 5:**
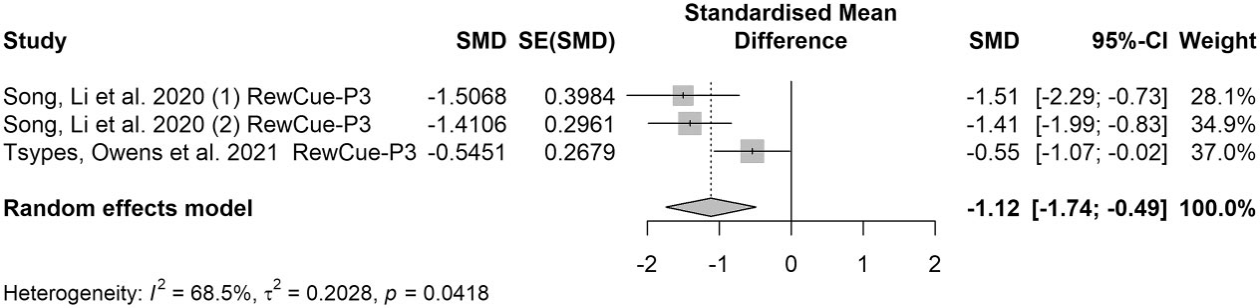
Forest plot of the reward-cue related P3 component in MID studies. Note: The elements in this figure are identical to those in Fig. 1 with a consistent representation of comparisons across multiple groups in Song *et al*.’s investigation.

In particular, P3 elicited by reward cues is common in reward-related tasks such as the MID task. The MID task typically involves a series of trials in which participants are shown cues signaling the potential for a reward. After the cue, there is a delay period, followed by the presentation of a target stimulus to which participants must respond quickly. Successful responses within a specific time window result in a monetary reward, while unsuccessful responses lead to no reward or even a monetary penalty. Notably, different from the go/no-go task emphasizing impulse control and response inhibition, the MID task focuses on reward processing and motivational anticipation. In contrast, the MID task reveals that a diminished P3 response to rewards reflects impairments in reward processing and engagement with positive stimuli. Across these studies, a consistent finding emerged—individuals with a history of SI and SA exhibited a blunted P3 in response to positive feedback, as shown in the meta-analysis of MID studies (SMD = −1.12, 95% CI = [−1.74; −0.49], see Fig. 5). The *I*² value of 65% suggests moderate to substantial heterogeneity among the studies. Egger's test revealed no publication bias existed (*b* = −6.58, *P* = 0.493, see Supplementary Fig 5).

FRN, temporally close to N2, is distinct in its function. In go/no-go tasks, suicidal individuals exhibit smaller FRN amplitudes in the no-go conditions, indicating difficulties in self-control and impulse inhibition that can impact their decision-making processes. Studies using the MID task have found that individuals with a history of SA show reduced FRN amplitudes in response to positive feedback, suggesting impaired processing of reward feedback. The ΔRewP typically occurs earlier than the P3 component, generally peaking around 250–350 ms post-stimulus, whereas the P3 peaks later, around 300–500 ms. Despite their close temporal proximity, the RewP and P3 reflect distinct aspects of reward processing, with RewP associated more directly with feedback evaluation and P3 linked to attentional and motivational processes. The ΔRewP, measured in tasks such as the guessing doors task, captures the differential processing of reward versus non-reward feedback and serves as a promising neurophysiological marker in suicide risk assessment. Evidence suggests that ΔRewP may serve as a transdiagnostic brain-based marker of SI, and blunted RewP exists in children with SI. However, FRN and ΔRewP were not included in the synthesis because of insufficient robustness. Studies utilized either principal component analysis (PCA) or traditional differential waveform techniques to analyze ERP components, resulting in discrepancies in reported amplitudes and latencies of components such as FRN and ΔRewP.

### The late-latency ERP component

**Figure 6:**
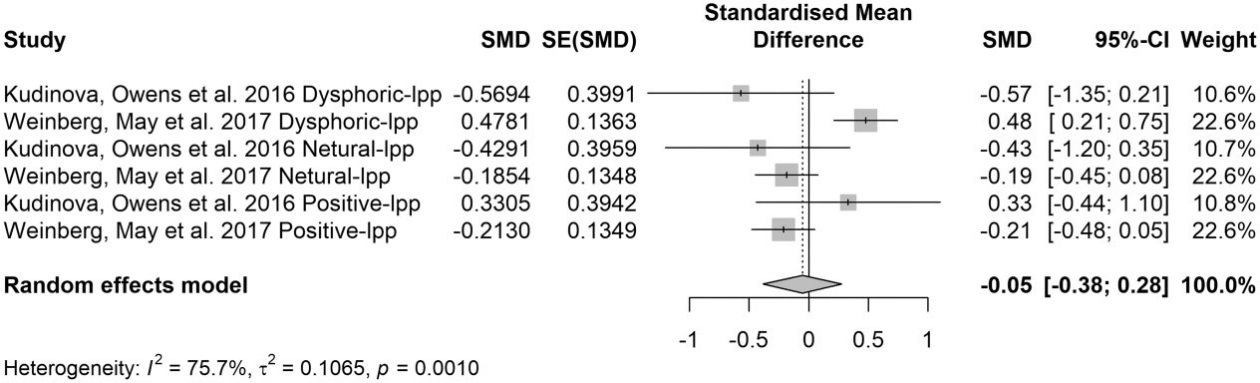
Forest plot of the late positive potential (LPP) in emotional image processing studies. Note: The elements in this figure are identical to those in Fig. 1 with additional details pertaining to the LPP condition.

LPP has indeed been widely studied in the context of emotional and motivational processing, its direct application to suicide risk is a more recent and emerging area of research. Few studies have utilized image-viewing tasks to examine neural responses to emotional stimuli in this context. However, different studies may employ varied experimental paradigms, which can influence the elicitation of LPP responses. The forest plot in Fig. 6 synthesizes results from two studies investigating LPP responses to emotional stimuli in individuals at risk of suicide. The results indicate that individuals with SA displayed varying LPP amplitudes in response to emotional stimuli compared to healthy controls. Due to the limited number of studies available, the analysis focused solely on comparing individuals with a history of SA to healthy controls. The *I*² value of 76% suggests moderate to substantial heterogeneity among the studies. Egger's test revealed no publication bias existed (*b* = −0.91, *P* = 0.681; see Supplementary Fig. 6).

### Qualities of studies

**Figure 7:**
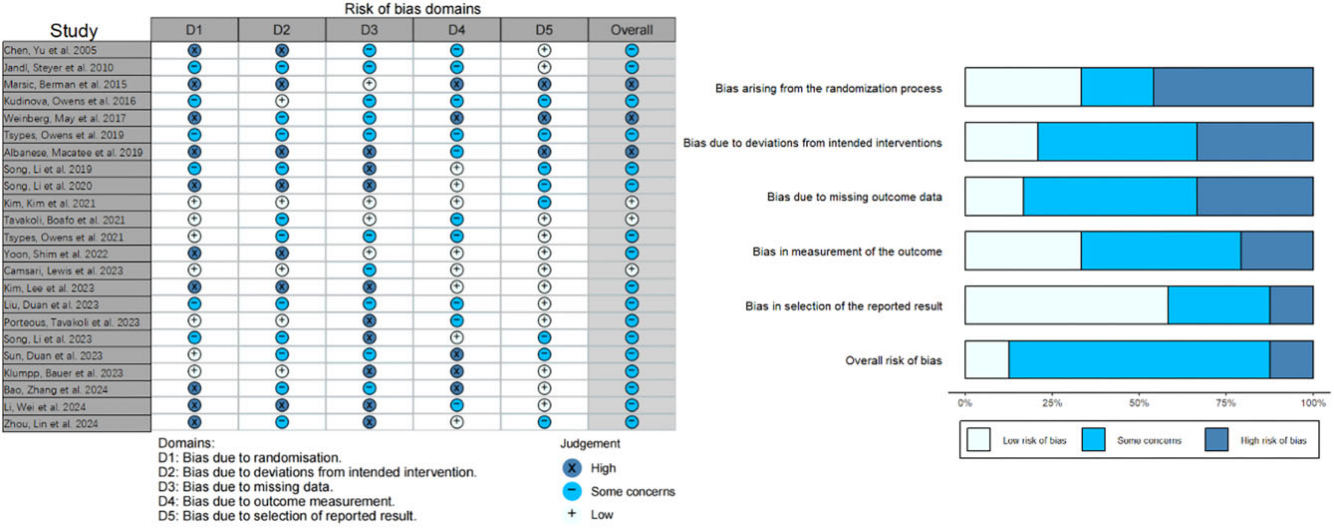
Assessment of risk of bias in meta-review studies. Note: The studies were organized based on their publication years, following the order presented in Tables 1 and 2.

Quality and reproducibility may be limited by the heterogeneity of the samples, differences in task design and stimuli, and the relatively small sample sizes in many of the studies. The meta-review results shown in Fig. 7 indicate varying levels of bias across different categories. The risk of bias assessment across 23 studies reveals variability across five domains. For bias due to randomization (D1), 30.4% of studies were at low risk, 21.7% had some concerns, and 47.8% were at high risk, indicating issues with randomization. For bias due to deviations from intended interventions (D2), 21.7% were at low risk, 43.5% had some concerns, and 34.8% were at high risk. The bias due to missing data (D3) showed 17.4% at low risk, 47.8% with some concerns, and 34.8% at high risk, while bias in outcome measurement (D4) had 34.8% at low risk and 43.4% with some concerns, with 21.7% at high risk. For bias due to selection of the reported result (D5), 56.5% were at low risk, 30.4% had some concerns, and 13.1% were at high risk. Overall, 13.1% of studies were assessed as having a low risk of bias, 73.9% had some concerns, and 13.0% were at high risk of bias. Hence, the prevalence of studies with “some concerns” about bias underscores the necessity for improved methodological rigor in forthcoming research endeavors to elevate the quality and credibility of the existing evidence base.

## Discussion

The current systematic review examined the use of ERPs to investigate the neural correlations of suicide risk, with a focus on distinguishing individuals at suicide risk from those without a history of SI and SA. A variety of tasks are employed in studies, with a predominant focus on attentional processing (e.g. oddball tasks), impulse control tasks (e.g. go/no-go task), emotion-related tasks (e.g. emotional Stroop task), and reward-related tasks (e.g. doors task, monetary reward task). The most frequently analyzed ERP components include LDAEP, N2, P3, and LPP. One of the key findings is the reduced P3 amplitude observed in individuals with a history of SA compared to those with SI and healthy controls. The review of available studies suggests that individuals with SI or a history of SA exhibit distinct patterns of neural activity compared to healthy controls, particularly in the context of emotional and cognitive processing.

### Neural correlates of suicide risk: impairments in attention and cognitive control

The diminished P3 component in individuals with a history of suicide risk reflects impairments in attention, inhibitory control, and decision-making processes (Kim *et al*., 2023; Zhou *et al*., 2024). In particular, it would contribute to the transition from SI to SA (Yoon *et al*., 2022). Our analysis of research utilizing the go/no-go task indicates a consistent disparity in the P3 component response observed during the no-go condition between individuals who have attempted suicide and those experiencing suicidal thoughts. Specifically, reduced P3 amplitudes may indicate challenges in directing attentional resources towards stimuli that hold motivational significance, such as emotional cues. Furthermore, investigations employing reward cue viewing tasks consistently report lower P3 amplitudes in individuals with a history of SA compared to those with SI alone (Song *et al*., 2020; Tsypes *et al*., 2021). This suggests that individuals who have previously attempted suicide may demonstrate impaired neural responses to positive feedback and a reduced ability to process and respond to rewarding stimuli, which could contribute to their increased risk of suicidal behaviors. Moreover, the findings indicate that the P3 component may be a more sensitive neurophysiological marker for distinguishing and monitoring individuals with a history of SA. However, in the same paradigms, the N2 component was not found to consistently distinguish between these two groups. Relatedly, as Tavakoli and colleagues explained, the enhanced N2 amplitudes observed in individuals at suicide risk suggest increased cognitive control and conflict detection processes, potentially reflecting a heightened sensitivity to emotional conflicts and a greater need to exert cognitive effort to resolve these conflicts (Tavakoli *et al*., 2021). However, Zhou *et al*. suggested that these discrepancies could be partly due to variations in sample size and sample population (Zhou *et al*., 2024). Taken together, these findings suggest that while attentional difficulties (reflected by reduced P3) appear to be a relatively consistent feature of suicide risk, the role of conflict monitoring (reflected by N2) is less clear. It is possible that N2 alterations are present in some individuals at risk of suicide but not others, or that the specific conditions under which N2 differences emerge have not yet been fully identified.

### Exploring early ERP components and serotonergic function in suicide risk

While the significant findings regarding the P3 component are noteworthy, the other ERP components also warrant further examination and discussion. Our literature review suggests that the connection between early ERP components and suicide risk remains unclear. We found that oddball tasks were commonly employed to investigate these early ERP components. Generally, the reduced amplitude of these early components may indicate impaired early attentional functioning, potentially related to task difficulty. However, in most of the included studies, the N1 component was only examined as part of the investigation of LDAEP, aiming to identify a non-invasive physiological marker through the relationship between LDAEP and serotonin levels in cerebrospinal fluid. The LDAEP has been used as a measure of central serotonergic function in various clinical populations, including depression, schizophrenia, and Parkinson's disease (Hagenmuller *et al*., 2016; Roser *et al*., 2020). Although prior research has suggested that individuals who die by suicide may exhibit disruptions in serotonin function, particularly in the context of depression, the current review did not find a consistent pattern of abnormalities in the loudness dependence of auditory evoked potential, which is commonly used as an indirect measure of serotonin activity, regarding suicide risk. The available evidence indicated that individuals who have attempted suicide showed lower LDAEP compared to non-attempters, but this finding was superficial and did not account for the potential psychological effects, as noted by Kim *et al*. (2021). Furthermore, there were variations in methodologies between the two studies reviewed for LDAEP, including discrepancies in experimental design, stimulus intensity, and auditory tasks. Variations in how the LDAEP is measured, such as differences in auditory stimulus parameters, can lead to diverse neuronal responses. While the present review found heterogeneous results concerning the relationship between LDAEP and suicide, continued research in this domain may help elucidate the role of serotonergic dysfunction in the neurobiology of suicide risk. However, based on our current review, the relationship between LDAEP and serotonin in suicide populations displays substantial variability, necessitating further exploration.

### The role of LPP in emotional processing and suicide risk

The last ERP component relevant to suicide risk is the LPP. Like P3, the LPP is modulated by emotional salience and can provide insights into the emotional processing and regulation of individuals at suicide risk (Kudinova *et al*., 2016; Song *et al*., 2023; Zhao *et al*., 2023). LPP is highly context-dependent, especially when tasks involve emotional stimuli. Variations in complexity, imagery type, and emotional content can impact how subjects process emotional information and affect LPP amplitudes. Factors such as participants' mood state, task familiarity, and experimental environment can also influence LPP responses. Inconsistently controlled contextual influences across studies may result in differing LPP outcomes. Therefore, meta-analysis of LPP varies more across studies in this review. Though fewer studies have examined the LPP in this context, the available evidence suggests that individuals with a history of SA may exhibit attenuated LPP amplitudes in response to emotional stimuli compared to those with SI alone (Kudinova *et al*., 2016; Weinberg *et al*., 2017). This may indicate impairments in the sustained emotional processing and regulation of affective information, contributing to the heightened risk for suicidal behavior. However, the LPP is also thought to reflect emotional regulation processes. A greater LPP change in emotional states may indicate difficulties in controlling and regulating emotional reactions, which could result in increased emotional responsiveness and impulsiveness. The studies examining the LPP in response to stimuli associated with life versus death revealed intriguing differences between healthy individuals and adolescents with a history of SA (Camsari *et al*., 2023). In the healthy group, larger LPP-like activity was observed during blocks containing life-congruent information compared to death-congruent blocks. In contrast, the suicide-risk group showed similar or slightly larger LPP-like activity during death-congruent blocks relative to life-congruent blocks. The pattern observed in the study by Camsari suggests a potential difference in how individuals at risk for suicide process and respond to information about life and death. However, the task used in Camsari *et al*.’s study differs from those analyzed in this review. It is important to clarify that their implicit association task assessed automatic cognitive associations between life- and death-related concepts rather than directly eliciting emotion regulation strategies. This distinction may underlie differences in LPP patterns observed across studies. Whereas the tasks reviewed typically involved explicit or passive emotional processing, Camsari *et al*.’s paradigm taps implicit associations, underscoring the need for future investigations to systematically explore LPP responses across a wider range of emotional and cognitive task contexts to better understand the neural correlates of suicidal behavior.

### Exploring ΔRewP, FRN, and their clinical implications

RewP and FRN are also frequently measured ERP components related to decision-making tasks and reward processing, respectively. ΔRewP signifies the variation in RewP between feedback of reward and no reward/loss receipt by participants (Hager *et al*., 2022). It is mostly measured by the doors task and differs from the reward cue-P3, as it measures the degree of cognitive control when receiving feedback on rewards (Wang *et al*., 2020). Two studies in this review that utilized the doors task found that children with SI exhibited significantly reduced variation in the reward positivity compared to children without SI (Tsypes *et al*., 2019; Klumpp *et al*., 2023). Despite employing various analysis approaches in these studies, they were inadequate for a meta-analysis. This indicates that impairments in reward processing and decreased responsiveness to positive feedback could be factors in the emergence of suicidal thoughts in young people (Bress *et al*., 2023). Considering the limited research on this, it is important for future research to continue exploring the role of reward processing in suicide risk. FRN was studied more extensively than RewP, as it could be measured in more types of the task, such as monetary loss tasks. In tasks involving monetary gains and losses, it has been observed that individuals with suicidal tendencies exhibit heightened neural responses to losses (Tsypes *et al*., 2021). Specifically, these individuals demonstrate greater feedback negativity or feedback-related negativity following monetary losses, indicating a heightened sensitivity to negative outcomes (Klumpp *et al*., 2023). This may be indicative of an overvaluation of negative emotional states or a tendency to ruminate on negative experiences, both of which are known risk factors for suicidal behavior. However, some research shows there is no difference between a suicide attempt group and healthy control group (Song *et al*., 2019). Due to its dependency on the task, a more complicated meta-analysis is needed if researchers want to find the relationship between them.

### Multimodal EEG features of suicide risk

Several studies have explored multimodal EEG features of suicide risk, combining different ERP components and analysis techniques to improve the accuracy of suicide ideation classification. For example, Song and colleagues examined behavioral and ERP features involved in pain processing as predictors of suicide ideation (Song *et al*., 2020, 2023). They assessed 27 depressed undergraduates with high suicide ideation, 23 depressed undergraduates with low suicide ideation, and 32 healthy controls using clinical scales. The researchers examined a range of multimodal EEG features, such as the amplitudes of LPP, P3, FRN, and other features, through time–frequency analysis during a task. Using a machine learning algorithm, they found that the accuracy of suicide ideation classification was significantly higher when considering multiple features, with pain avoidance ranked as the most important feature, followed by contingent negative variation of ERP. This suggests that pain avoidance and its related EEG features may be more effective in classifying suicide ideation compared to using depression alone. Furthermore, integrating advanced machine learning algorithms with these neurophysiological markers could potentially develop more sophisticated predictive models. By analyzing complex interactions between brain activity patterns, psychological profiles, and clinical indicators, researchers might create more nuanced and personalized suicide risk assessment tools that offer earlier and more precise interventions.

### Limitations and strengths

The limited number of ERP-based studies on suicide risk hinders the ability to draw definitive conclusions compared to functional magentic resonance imaging, positron emission tomography, and neurochemical research. Therefore, the included studies in this review may not be enough for interpretability of the funnel plots. Although we conducted a comprehensive literature search using general ERP-related terms, limitations inherent to keyword-based and database search strategies may have resulted in the unintentional exclusion of studies employing specialized or less common terminology. Moreover, the heterogeneity of the included studies in terms of study design, clinical populations, and specific ERP components examined poses a challenge. Future studies should aim to distinguish between SI, SA, and completed suicides in order to identify specific neural correlates associated with each stage of suicidal behavior. More refined methodologies are needed to differentiate the roles of ERP components in these categories. Furthermore, the predominance of cross-sectional designs in the literature makes it challenging to establish causal relationships between ERP abnormalities and suicidal behaviors. Future longitudinal studies with larger, more homogeneous samples are necessary to clarify the role of ERPs in suicide risk assessment and to develop objective neurophysiological-based tools for suicide prevention. Despite these limitations, the review suggests that certain ERP components, especially the P3, show promise as biomarkers for assessing suicide risk.

## Conclusion

This systematic review provides a comprehensive overview of the existing literature on the use of ERP in the detection and assessment of suicide ideation and SA. The available evidence suggests that specific ERP components, particularly the P3, may serve as a promising indicator for evaluating suicide risk. Individuals predisposed to suicide risk show changes in P3 amplitudes, indicating deficits in inhibitory control, attention, and processing of reward cues. Future longitudinal studies with larger, more homogeneous samples are needed to further elucidate the role of ERPs in suicide risk assessment and to inform the development of objective, neurophysiological-based tools for suicide prevent.

## Supplementary Material

kkaf018_Supplemental_File
