# Supplementary material for: Detection of suicide risk using event-related potentials: a comprehensive systematic review and meta-analysis
Source: Psychoradiology. 2025 Jun 11;5:kkaf018. doi: 10.1093/psyrad/kkaf018 (PMC12205307; doi:10.1093/psyrad/kkaf018)
Supplement: kkaf018_Supplemental_File [file kkaf018_supplemental_file.docx]

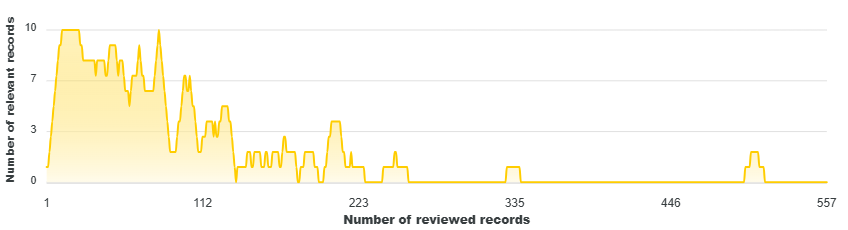


**Supplementary figure 1: ASReviewLAB progress Density**


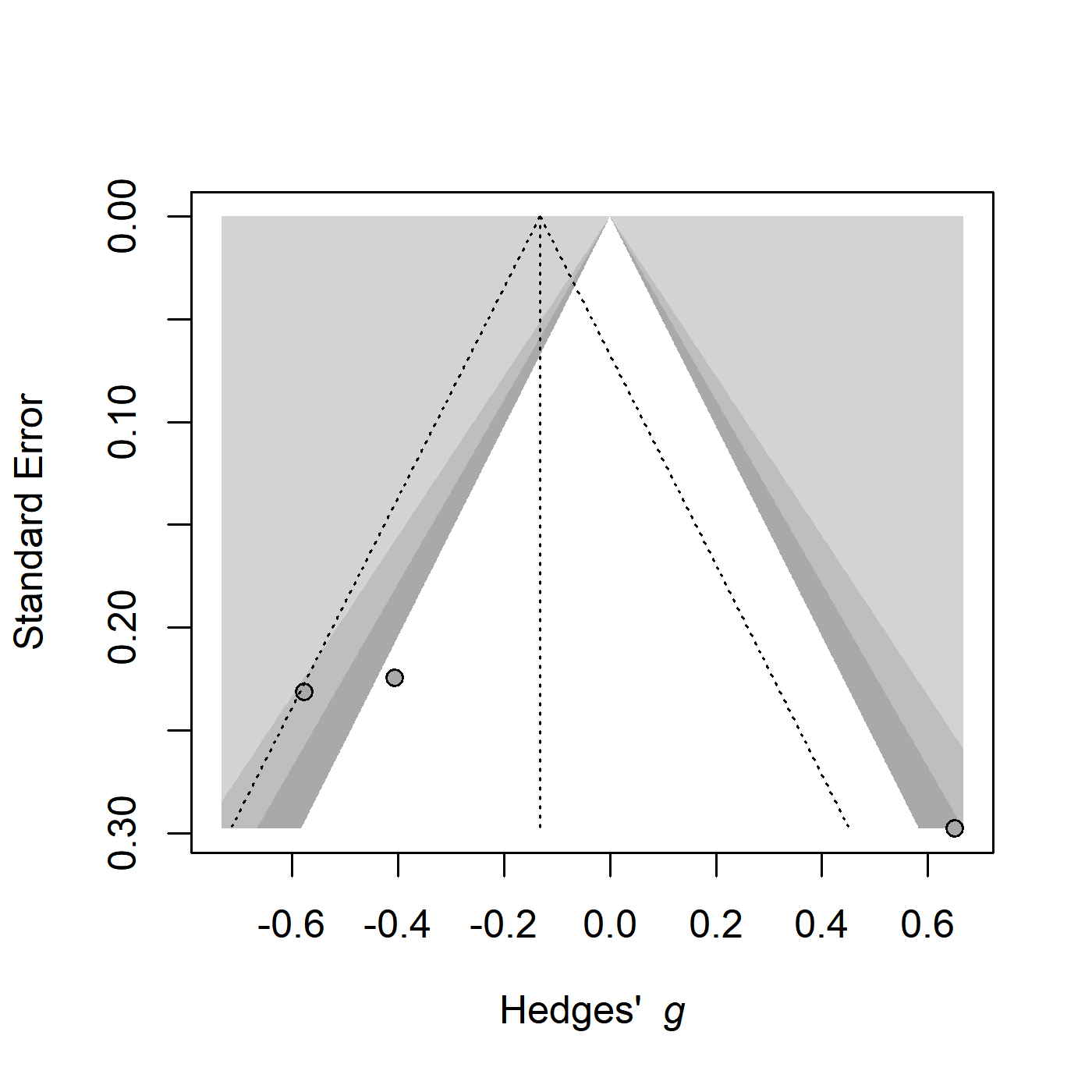


**Supplementary figure 2: funnel plots for LDAEP**

Note: X-Axis indicates the range of effect sizes, with a reference line for the summary effect size. Y-Axis indicates that smaller studies are at the bottom and larger studies are at the top. The Vertical Dotted Line indicates that this line represents the overall effect size across studies. Gray Contours highlight that the areas in gray represent significant effect sizes, particularly among smaller studies. The studies (located at the bottom of the funnel) shows statistically significant effect sizes, indicated by being within the gray contours. This suggests that these studies report effects that are meaningful and not due to random chance.


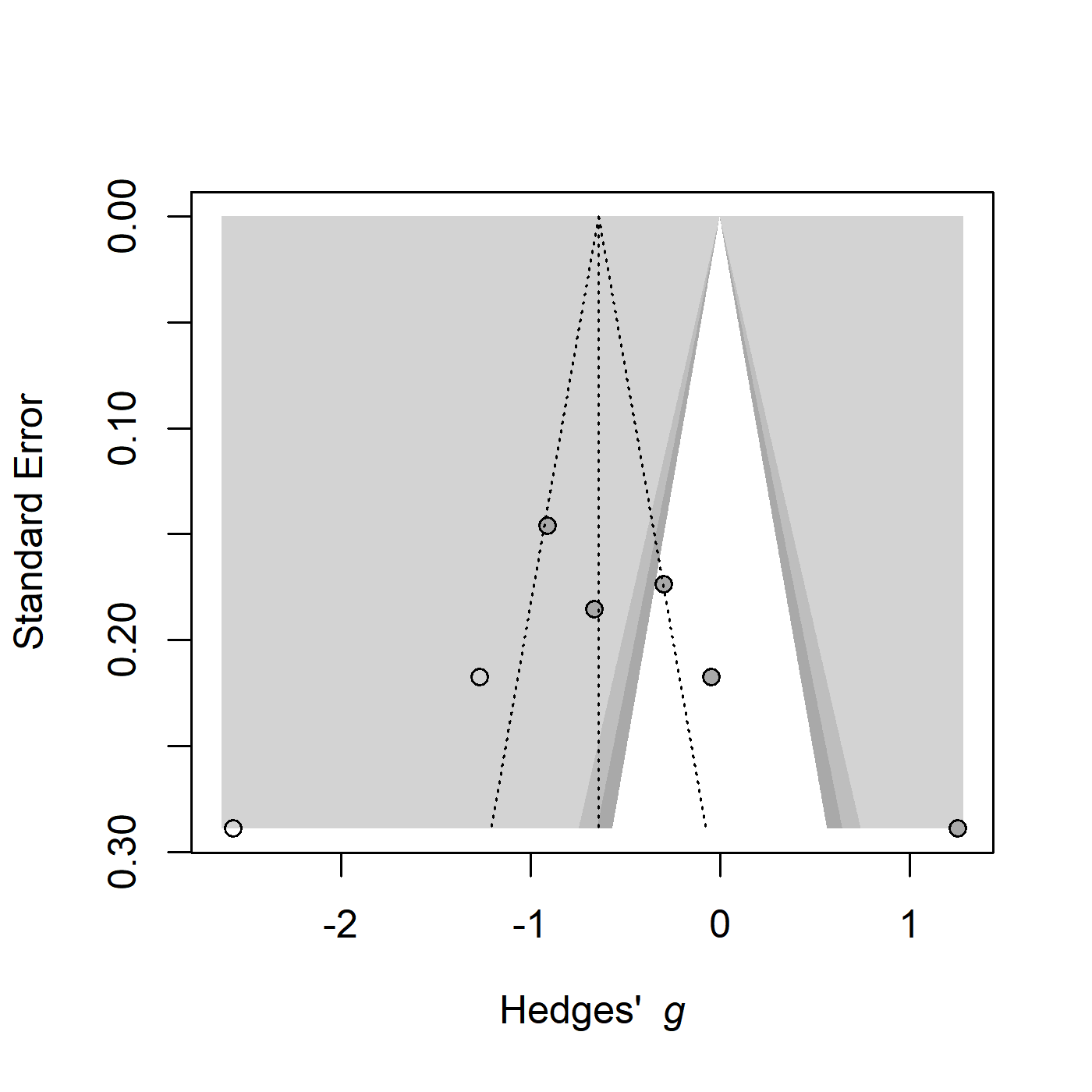


**Supplementary figure 3: funnel plots for N2**

Note: The funnel plot shows asymmetry, particularly with smaller studies concentrated on the left side (negative effect sizes) and fewer studies appearing on the right side (positive effect sizes). This implies that there might be a publication bias, as smaller studies with nonsignificant findings are less likely to be published, potentially affecting the overall conclusions drawn from the research in N2.

**
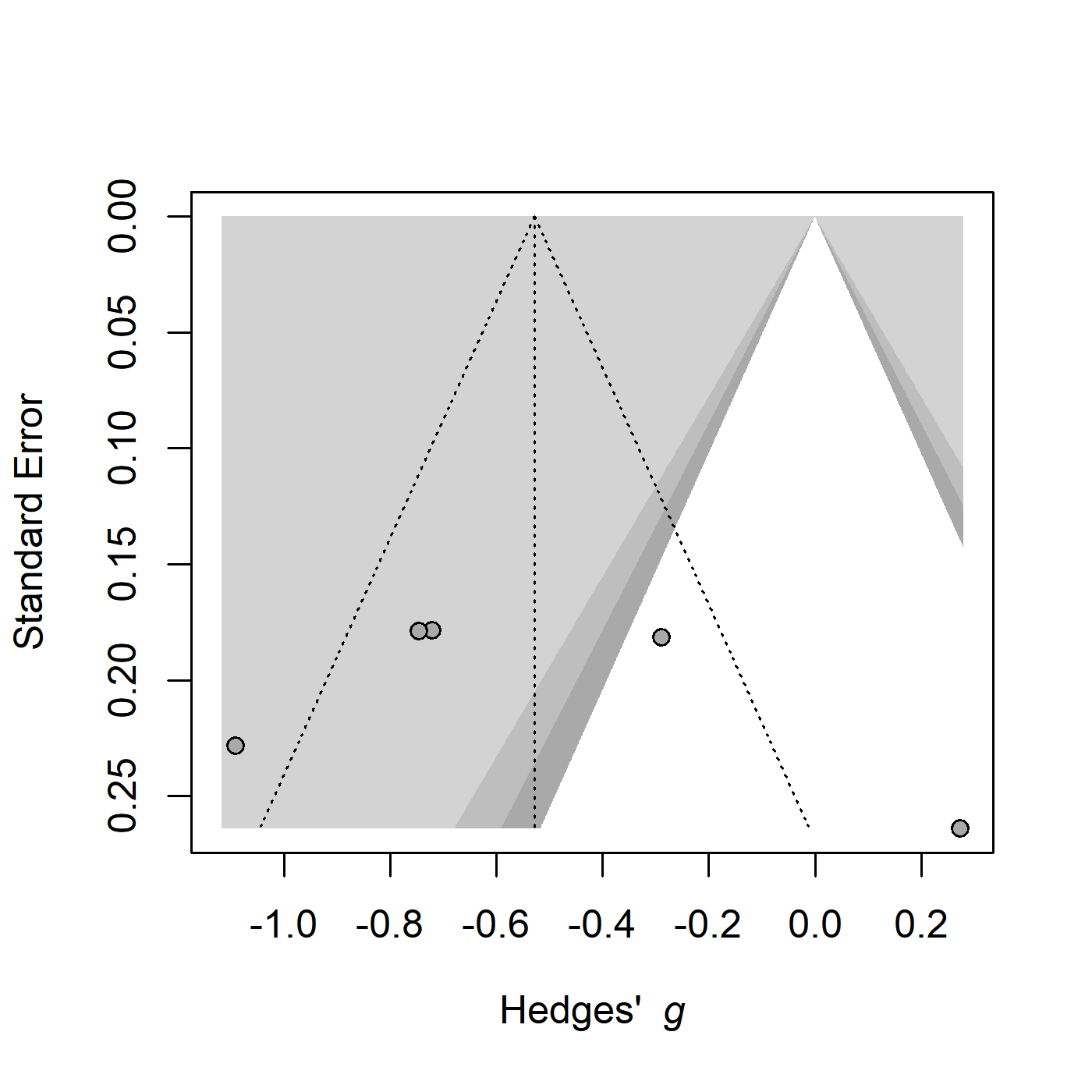
**

**Supplementary figure 4: funnel plots for P3**

Note: The funnel plot exhibits a noticeable asymmetry, with a greater concentration of studies reporting negative effect sizes (to the left of the vertical line at zero) compared to those reporting positive effects (to the right). Many of the studies located within the shaded region report negative effects, indicating that these studies found meaningful outcomes favoring the group associated with negative Hedges' g values. In contrast, fewer studies with positive effect sizes are present, particularly in the nonsignificant region. The predominance of negative effect sizes among the studies raises concerns regarding the completeness of the published literature. It suggests that significant negative findings may be favored, while studies with nonsignificant results, particularly those with positive effects, might be missing from the analysis.


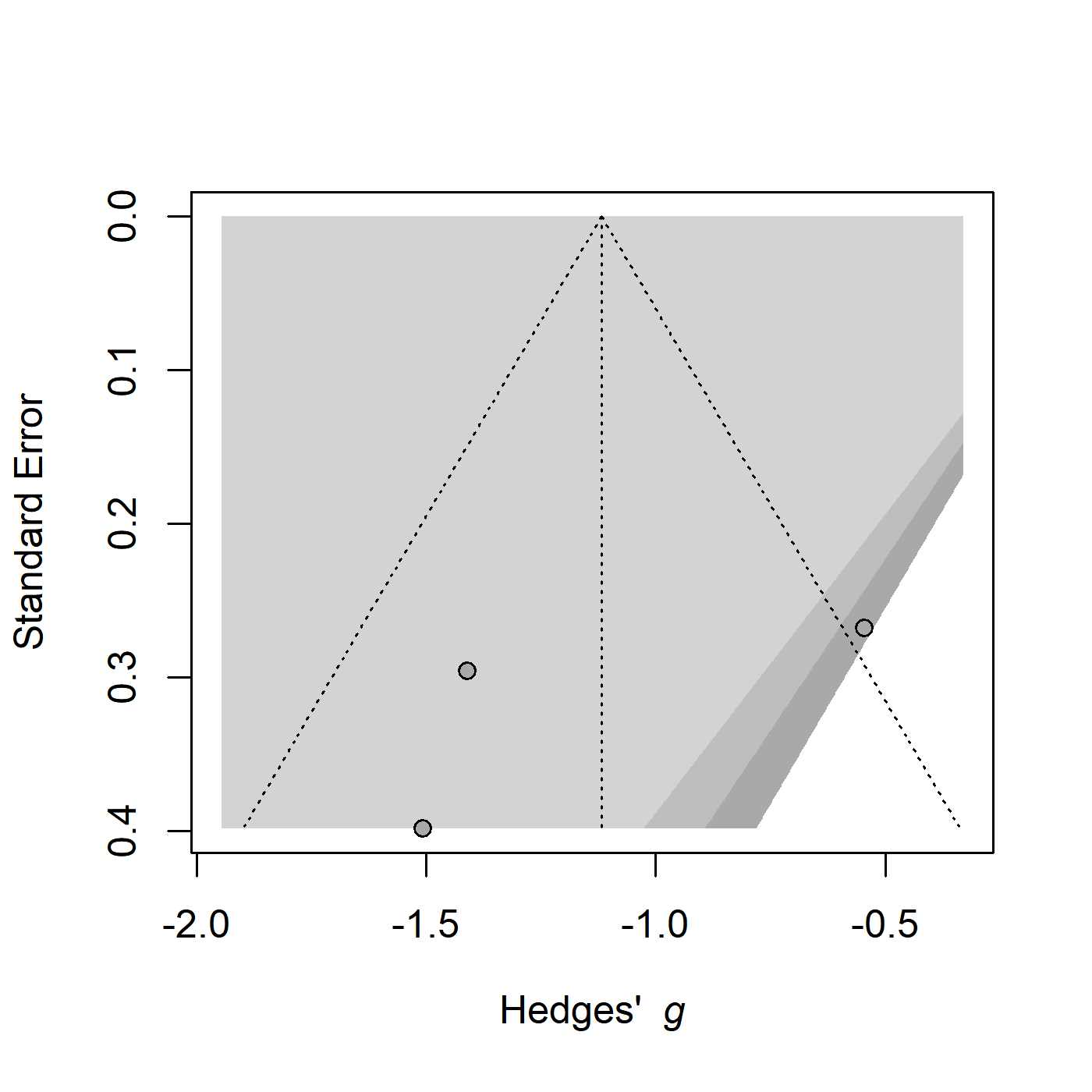


**Supplementary figure 5: funnel plots for Rew-cue P3**

Note: The funnel plot appears asymmetrical, particularly with fewer studies positioned on the less negative side of the effect size spectrum. This asymmetry raises concerns about potential publication bias, suggesting that studies with nonsignificant or positive results might be underrepresented in the literature. The funnel plot also indicates a predominance of studies reporting significant negative effects of the suicide risk, with potential publication bias suggesting that nonsignificant findings may be underreported. The implications of these results highlight the importance of considering the completeness of the literature when interpreting the effects being studied.


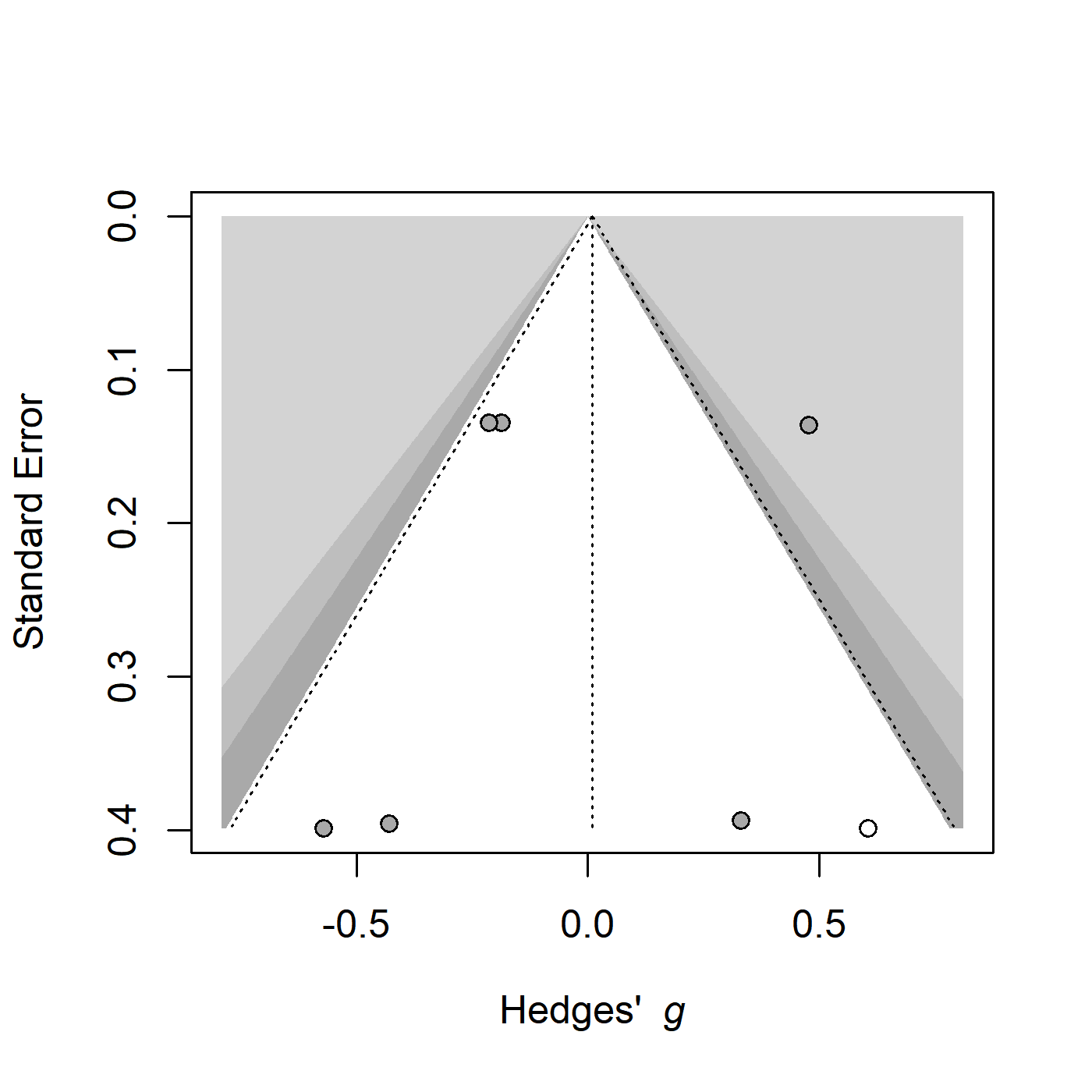


**Supplementary figure 6: funnel plots for LPP**

Note: In summary, the funnel plot indicates a balanced distribution of studies with a mix of positive, negative, and nonsignificant effect sizes. The relative symmetry of the plot suggests minimal publication bias, allowing for a more comprehensive understanding of the effects being studied. This highlights the need to consider both significant and nonsignificant findings in interpreting the overall results.
